# Evaluation of oral health in 148 patients with systemic sclerosis—data from a prospective interdisciplinary monocentric cohort

**DOI:** 10.1007/s00296-024-05635-z

**Published:** 2024-06-14

**Authors:** Ann-Christin Pecher, Bahar Günaydin, Hannah Finke, Jörg Henes

**Affiliations:** 1 Interdisciplinary Center of Rheumatic Diseases (INDIRA), University Hospital Tuebingen, Tuebingen, Germany; 2 Eberhard Karls University of Tuebingen, Tuebingen, Germany; 3 Department of Orthodontics, University Hospital Tuebingen, Osianderstr. 2-8, 72076 Tuebingen, Germany

**Keywords:** Systemic sclerosis, Dental hygiene, Periodontitis, Oral manifestations, Surveys and questionnaires

## Abstract

In daily rheumatology practice, systemic sclerosis is primarily regarded as a potentially life-threatening disease characterized by fibrosis of various organs. Therefore, other manifestations, such as orofacial involvement, are often not of primary concern. Furthermore, due to its rarity, the disease might not be well known by dentists, which contrasts with the increased risk of various problems in the oral cavity. Periodontitis in particular is a known risk factor for morbidity and mortality and is associated with various systemic diseases. The risk of periodontitis appears to be increased in patients with systemic sclerosis, but little is known about the gender-specific differences. This study aims to elucidate the health-conscious behaviour of patients, their dental care and the risk of periodontitis with regard to gender-specific differences. This descriptive study of the Interdisciplinary Centre of Rheumatic Diseases (INDIRA) in collaboration with the Department of Orthodontics at the University Hospital of Tuebingen, Germany, examined the data of 148 patients with systemic sclerosis with regard to their oral health using a questionnaire and evaluating the risk of periodontitis with the DG Paro self-assessment score in this cohort. Among the participating patients, 90% reported regular visits to the dentist and good dental care. Nevertheless, more than half of the patients had missing teeth and problems opening their mouths. Sicca symptoms in the oral cavity were also common (40%). The risk of periodontitis among female participants was high (around 60%), and even higher among male study participants (around 80%). Gingival bleeding as a surrogate parameter for periodontitis was associated with salivary flow and the modified Rodnan skin score (mRSS). Despite a high awareness of dental health, we observed a high risk of periodontitis, especially in male patients with systemic sclerosis. In addition, the association between xerostomia and missing teeth as well as gingival bleeding and mRSS may indicate an increased risk in patients with a more progressive disease. We would therefore recommend regular dental consultations and careful oral hygiene for patients with systemic sclerosis in addition to the—more organ-focused—regular examinations of patients.

**Supplementary Information:**

The online version contains supplementary material available at 10.1007/s00296-024-05635-z.

## Introduction

Systemic sclerosis (SSc) is a potentially life-threatening systemic disease with typical manifestations such as fibrosis of the skin and internal organs. SSc is generally associated with typical autoantibodies against topoisomerase-I (Scl70), centromere (ACA) or RNA polymerase III among others. Furthermore, Raynaud’s syndrome and other clinical manifestations of vasculopathy are present. The incidence varies regionally and is approximately 0.5–2/100,000 individuals and typically affects women beyond the 5th decade [1].

Skin fibrosis can also affect the facial skin, and numerous manifestations in the oral cavity are possible [2–5]. Furthermore, SSc is often associated with xerostomia and hyposalivation with a certain predisposition to caries and periodontitis, as is also known for Sjogren's syndrome [6–8]. Due to frequently present microstomia and/or reduced mouth opening, dental hygiene and dental care by the patient and by the dentist may be restricted. Another problem is the use of immunosuppressive drugs for the treatment of SSc—e.g. methotrexate and cyclophosphamide—which can lead to changes in the oral cavity in the form of mucositis and thus cause secondary gingival atrophy or ulceration. Particularly in patients with SSc malnutrition might also be present and thereby additionally affect (oral) health.

Periodontal disease is an inflammation of the gingiva and the periodontal tissues. It arises from prolonged biofilm formation in hand with an immunologic answer towards the bacterial load. Clinical attachment loss (CAL) describes an increasing destruction of the periodontium in the context of periodontitis. This can result in detachment of the gingiva from the tooth and damage to the periodontal ligament including the alveolar bone. In consequence, the tooth loosens and finally gets lost [9]. Thus, periodontal disease not only affects comfort, but also nutritional and aesthetic aspects. Furthermore, due to chronic inflammation, periodontitis is associated with several other systemic conditions such as diabetes, cardiovascular disease, pregnancy complications and cancerogenesis amongst others [10–13].

In clinical practice, there is little exchange between the treating rheumatologists and dentists. In addition, many SSc patients have difficulty finding a dentist with knowledge of their (admittedly rare) disease [3]. Even though 80% of the patients suffer from orofacial involvement, there is currently little data on the risk of periodontal disease in this group. Overall, oral health-related quality of life is significantly reduced in patients [2, 3]. Despite their frequency, orofacial manifestations are underestimated and insufficiently investigated, as the focus tends to be on the life-threatening involvement of internal organs [14].

## Methods

### Study design

This interdisciplinary observational monocentric study on oral health in patients with SSc was conducted at the University Hospital Tuebingen, Germany, in cooperation with the Department of Internal Medicine II (haematology, oncology, immunology and rheumatology) and the Department of Orthodontics from 2018 until 2021.

### Patients

Patients were eligible if they were diagnosed with SSc according to the American College of Rheumatology/ European League Against Rheumatism criteria [15] and were at least 18 years old. During the survey period, every presenting patient at our department was asked to complete the questionnaire. Female participants were excluded if they were pregnant at the study visit. Questionnaires used in this study were completed by study participants during the consultation appointments or via mail.

### Questionnaire

The periodontitis risk score of this study was used according to the model of the German Society of Periodontology (DG PARO) in cooperation with the University of Greifswald [16–18], which is based on data from the German population survey Study of Health in Pomerania (SHIP) and validated in this cohort [19, 20]. It reflects the risk of periodontitis on self-reported variables and is interpreted as follows: 0–4 points = 4–23%, 5–6 points = 33–45%, 7–8 points = 58–69%, 9–10 points = 79–86%, 11–20 points = 91–100%. These data were used to calculate the periodontitis risk in this patient cohort.

We used all items from the DG PARO score, but the self-administered questionnaire (variables and results, refer to supplementary S1) also included variables associated with periodontitis and known SSc-related difficulties for oral health as follows: frequency of alcohol consumption, other diseases (diabetes, malignancy, food intolerance, dyslipidaemia), regular visits to the dentist, and routine dental hygiene measures (self and professional tooth cleaning, use of an electric toothbrush, interdental devices and mouthwash), number of teeth (based on no missing teeth = 28 teeth, self-reported), problems with dental hygiene (mouth opening, holding the toothbrush, pain, temporomandibular joint disorder, dry mouth), dietary difficulties (drinking while eating, difficulty swallowing food) and impairment due to problems with dental hygiene, xerostomia or oral health.

Disease parameters as modified Rodnan skin score (mRSS), disease duration, antibody status and medication were obtained from medical records.

### Ethical statement

The study was approved by the local ethics committee of the Eberhard-Karls-University Tuebingen (IRB approval number: 790/2018BO2, November 15th, 2018) to be in accordance with the ethical standards and with the Helsinki Declaration. All patients gave their written consent.

### Statistical analysis

Statistical analysis was performed using GraphPad Prism version 10 and SPSS IBM Corp version 28. Variables were compared between cases and controls using the χ2 test or Fisher exact test (two-tailed) for categorical variables and using the Mann–Whitney test for most continuous variables, and the t-test for variables that showed normal distribution in the Shapiro–Wilk test. P < 0.05 was considered statistically significant. When using multiple regression, B refers to the unstandardized and β to the standardized regression coefficient.

## Results

### Patients characteristics

A total of 148 patients were included (60% limited cutaneous SSc, 39% diffuse cutaneous SSc, 1% others), of which *n* = 111 were female patients and showed a median age of 53 years (min–max: 21–83 years). We registered a significantly higher proportion of female patients who never smoked (66% versus 44%, *p* < 0.05). Age, disease duration (median 6 years, min–max: 0–34 years), body-mass-index (BMI), SSA or SSB positivity (*n* = 14), level of education (54% at least 10 years of education) and alcohol intake (42% reported no alcohol intake at all, 43% occasionally, and 12% at least 2 × per week) were comparable in between groups.

### Dental status

Missing teeth were found in 59.5% of our study participants, gingival bleeding in 43.9% and tooth mobility in 16.9%.

**Fig. 1:**
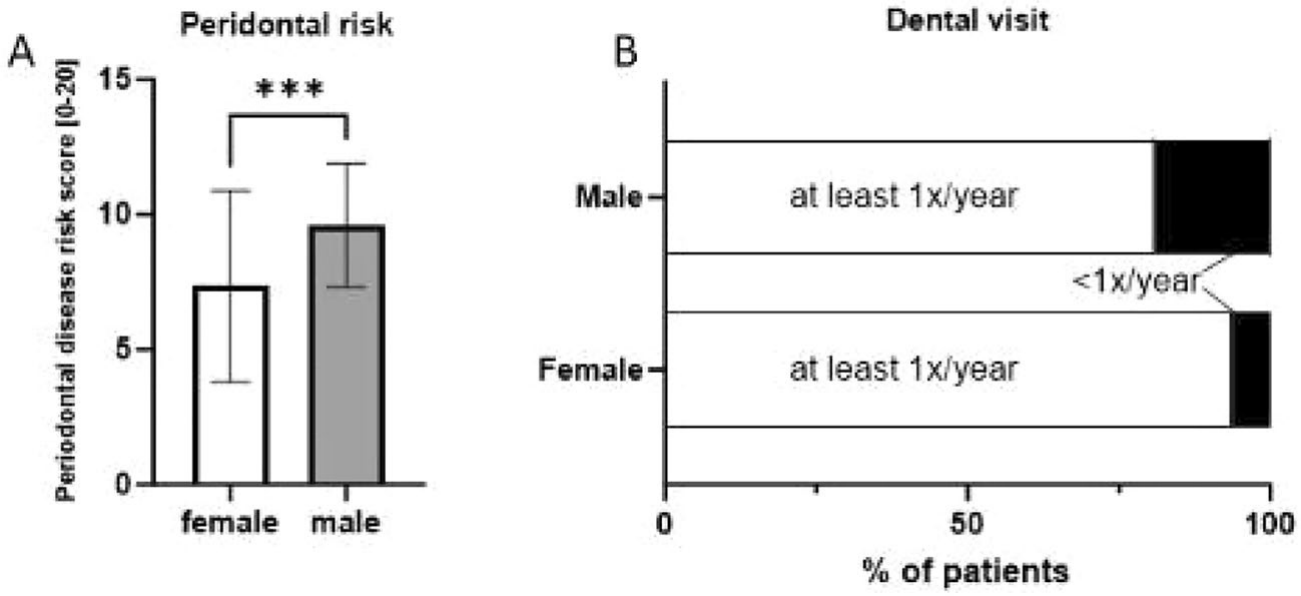
Comparison of female (*n* = 111) and male patients (*n* = 37) with systemic sclerosis. **A** Male patients showed a higher risk for periodontal disease (*p* < 0.001) using the self-reported questionnaire from the German Society of Periodontology and **B** the number of patients visiting the dentist at least once per year compared to never or only in case of complaints was also significantly lower in the male group (*p* < 0.05)

In this cohort male (M) patients showed significantly higher scores on the periodontitis risk score with a mean score of 9.6 ± 2.28 points (risk 79–86%) versus female (F) patients mean 7.34 ± 3.54 points (risk 58–69%), as illustrated in Fig. 1A.

### Dental care and associated challenges due to SSc

The majority of patients (F: *n* = 70, 63%, M: *n* = 18, 46%) visited their dentist twice a year. Only a few participants never attended the dentist or only when they had complaints (F: *n* = 7, 7%, M: *n* = 7, 19%). We noticed a significant difference between patients with regular dental care (at least 1x/year versus not at all/in case of complaints only) regarding gender (*p* < 0.05, Fig. 1B). Among the patients, 89% (*n* = 131) reported good dental care. However, only about one-third (*n* = 49) of the patients reported that their dentist had sufficient experience with SSc.

Self-cleaning was performed at least twice daily by most patients (F: n = 100, 90%, M: n = 27, 71%). We observed no strong gender-specific differences in the execution of professional dental cleaning. Within the patient cohort, 29% (*n* = 43) did not have their teeth professionally cleaned.

A self-reported smaller mouth opening was found in *n* = 67 (43%) of the test subjects. About one-third of the participants did not use interdental care devices (*n* = 51), mostly attributed to both, reduced mouth opening and dexterity (20%), and an additional 10% (*n* = 15) / 15% (*n* = 22) reported reduced mouth opening / reduced dexterity as the only cause for preventing the use of interdental care devices. The influence on tooth brushing was less prominent, only *n* = 22 (15%) reported dexterity, *n* = 10 (7%) (joint) pain and *n* = 11 (7%) both as causes which impeded tooth cleaning.

**Fig. 2:**
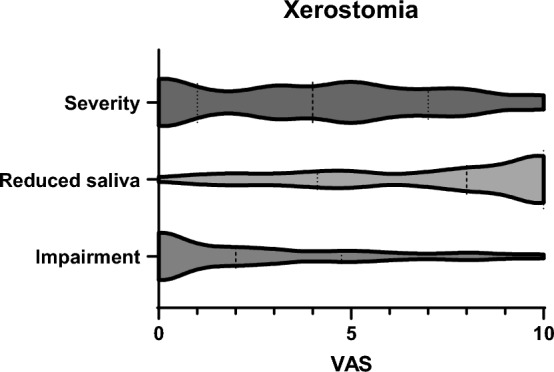
Violin plot, illustrating patient-reported outcome on the severity of xerostomia, reduced salivary flow and impairment of oral hygiene through xerostomia on a visual analogue scale (VAS) [0 = no xerostomia/no saliva/no impairment to 10 = very strong xerostomia/normal amount of salivary flow /severe impairment in daily oral hygiene]. Dashed lines report median and dotted lines quartiles

Xerostomia was frequently present, and affected *n* = 62 of the patients (F: n = 49, 44%; M: n = 13, 35%). Patient-reported outcome (Fig. 2) showed a high disease burden (visual analogue scale [VAS] median 4, [min–max 0–10]) for the severity of xerostomia manifestation and median 8 (min–max 0–10) for reduction of salivary flow), with however low clinical impairment on oral hygiene [median 3 (min–max 0–10)].

### Association of dental problems with disease and patients’ factors

Linear regression analysis (Table 1) was performed to estimate the relationship between the dependent variable number of missing teeth and various predictor variables concerning patient and disease characteristics, immunosuppressive medication and xerostomia. The number of pack years (B = 0.14, *p* < 0.05) and age (B = 0.29, *p* < 0.001) showed a significant relationship. For the dependent variable gingival bleeding only age (B = 0.03, *p* < 0.05), reduced salivary flow (B = −0.13, *p* < 0.05) and mRSS (B = −0.43, *p* < 0.05) were significant. We also found a significant relationship between xerostomia [VAS] and smoking (B = −0.04, *p* < 0.05), and age (B = 0.06, *p* < 0.05). As expected there was a significant relationship between mouth opening and mRSS with anti-Scl70 antibody status (B = −0.421, *p* < 0.05/B = 4.560, *p* < 0.01; data not shown) and xerostomia with SSA/SSB antibody status (B = 2.30, *p* < 0.05).

**Table 1:** Regression analysis for dental and oral abnormalities with sociodemographic factors, dental hygiene, disease parameters and medication

	Missing teeth†		Gingival bleeding‡		Xerostomia	
	B	p	B	p	B	p
Patient						
Age (years)	0.287	<0.001*; n = 134	0.031	0.014; n = 147	0.057	0.011*; n = 106
Gender	0.564	0.765	0.504	0.201	0.7	0.344
Pack years	0.14	0.016*; n = 121	0.006	0.634	−0.04	0.049*; n = 97
Education (at least 10 years)	−4.922	0.003*; n = 132	−0.232	0.488	0.2	0.747
Consultation dentist (at least 1x/year)	0.089	0.971	−0.506	0.318	−1.2	0.25
Brushing (at least 2x/day)	2.112	0.374	0.365	0.472	0.962	0.301
Autoantibodies						
ACA	0.415	0.829	−0.351	0.367	−0.008	0.991
Scl70	−1.959	0.231	0.561	0.094	−0.082	0.897
RNA Pol III	−5.197	0.228	−1.188	0.293	−2.175	0.237
SSA/SSB	4.134	0.148	−0.113	0.853	2.303	0.043*; n = 106
Xerostomia (VAS)						
Intensity	0.754	0.015*; n = 101	0.112	0.081	−	−
Reduced salivary flow	0.321	0.263	−0.126	0.033*; n = 128		
Medication						
Glucocorticoids	1.278	0.516	−0.235	0.559	0.196	0.78
Methotrexate	−2.462	0.24	0.095	0.827	−0.779	0.327
Cyclophosphamide	−1.962	0.683	1.366	0.242	−1.489	0.419
Mycophenolate	2.495	0.183	0.053	0.89	0.844	0.233
Disease characteristics						
Disease duration	0.002	0.836	−0.004	0.61	0.000	0.912
mRSS	−0.164	0.107	0.427	0.028*; n = 146	−0.024	0.557
Mouth opening (cm)	0.89	0.314	−0.018	0.398	−0.179	0.598
DUC	−0.403	0.55	0.046	0.748	−0.036	0.884

*significant, i.e. *p* < 0.05

**†** linear regression.

‡ binominal regression.

## Discussion

Oral involvement in SSc is common and concerns the quality of life of those affected. This study used questionnaires to record the oral manifestations of SSc and a self-assessment score from the German Society of Periodontology [17, 18] in a monocentric cohort of 148 patients in collaboration with the Department of Internal Medicine and Department of Orthodontics at the University Hospital Tuebingen.

Overall, the study participants had a high risk of periodontitis with male patients in particular having a significantly higher risk (female participants 58–69%; male study participants 79–86% risk for periodontal disease, p < 0.001). Also, over 50% of the study participants reported missing teeth—which has been shown to reflect clinical status of periodontal disease [21]. The higher risk in the male cohort may be due to lower health awareness among our male participants, who showed a tendency to increased smoking and drinking behaviour as well as less frequent visits to the dentist and poorer oral hygiene. However, sexual dimorphism in periodontal disease is also observed in the general population, and has been confirmed by various studies as highlighted by a review from 2010 [23]. The high prevalence of periodontitis in patients with SSc compared to healthy controls has also been suggested by previous studies [2, 22], however not confirmed by all [24]. There is evidence, that periodontitis is more common in patients with autoinflammatory diseases in general, thereby suggesting a link between autoimmunity and periodontal disease [2, 25–27]. So far, the mechanism has not yet been completely understood, beside the use of immunosuppressive drugs, other factors as osteoclastic bone damage, impaired oral hygiene, and proinflammatory cytokines have been discussed. There is evidence of elevated tumor necrosis factor-alpha (TNFα) levels and other proinflammatory cytokines in gingival crevicular fluid [28, 29] which might promote inflammation and consequently might lead to the destruction of periodontal tissue. Similarly, periodontal bone loss is associated with periodontitis, and higher levels of receptor activator of NF-kB ligand (RANKL) in the gingival crevicular fluid are associated with more severe disease but are also known to be present in patients with SSc, thereby offering a potential link in between both conditions [30, 31]. Tissue destruction might be further aggravated by vascular dysfunction—on of the hallmark pathologies in SSc—where microvascular damage is characterized by endothelial damage, perivascular inflammation and tissue hypoxia [32, 33].

Despite the high risk of periodontal disease, the awareness of oral health in our cohort appears to be high. Approximately 90% of the study participants went to the dentist regularly (2x/year: 60%, 1x/year: 28%) and affirmed good dental care. Nevertheless, almost 50% of the patients stated that their dentist had little experience with SSc. Toothbrushing was performed at least twice daily—as recommend by dental associations—by most patients (F: n = 100, 90%, M: n = 27, 71%). Other procedures as the usage of mouthwash, of interdental devices and professional tooth cleaning as recommended were used less frequently by only about one-third of the patients.

In our study, disease burden showed an impact on oral hygiene: More than half of the participants reported problems opening their mouths, which is caused by sclerosis of the lips and the skin around the mouth area which leads to microstomia and rhagades, Interestingly, VAS showed only a relatively low score (2.6 ± 2.9) for impairment with regard to dental and oral hygiene. Approximately one-third of the respondents had pain when opening the mouth and chewing, facial pain or blockages of the temporomandibular joint and mouth opening. Around 40% of patients reported xerostomia (VAS 4,5 ± 5.1). Thus, although the effect on oral manifestations seems to be perceived, other manifestations of the disease are most likely prioritized, as was also shown in the comparative questions on other complaints. It has to mentioned, that reduced mouth opening might cause impaired assessment and treatment of periodontitis and other oral pathologies. Therefore, a preventive approach is critical to avoid complications in our patients.. Furthermore, our data support the theory of a higher periodontal risk in patients with more severe skin manifestation as shown by other authors [27, 34], as we found a relationship between gingival bleeding and mRSS. We also identified reduced salivary flow as a risk factors for gingival bleeding as a surrogate parameter for periodontitis. In contrast to Baron [34] however, the number of missing teeth was not associated with decreased salivary flow, which has also been linked to an elevated risk of caries [35, 36]. In conclusion, the association in between disease severity and perio cannot be answered at the present time due to inconsistent findings [35]. Interestingly, disease duration and medication showed no significant relationship to oral health in our cohort.

There are some limitations to this study. First, we only relied on patients’ reported oral health parameters and did not perform an additional standardized examination in all of our patients. Even though the self-reported oral health variables have been shown to be valid and reflect clinical examination [21], clinical examination would have been desirable. Furthermore, we did not involve a control group to evaluate the periodontitis risk compared to other autoimmune diseases or healthy controls, as our questionnaire focussed on SSc-related risk factors. Finally, even though the DG-PARO self-assessment score has been validated in a general German population, the whole questionnaire has to be validated yet on patients with SSc.

Taken together, our results confirm the abundant problems that SSc patients confront with dental hygiene and the association of disease severity (i.e. mRSS and salivary flow) with oral health. Despite the more threatening organ manifestations, oral health problems in these patients should not be neglected and not only physicians but also patients should be made aware of this important health issue. As our data suggest, not only disease specific parameters, but also established parameters such as smoking, education, and age are important risk factors, patients should be encouraged to have regular dental examinations and an oral health protective lifestyle and treatment.

### Supplementary Information

Below is the link to the electronic supplementary material.

Supplementary file1 (DOCX 20 KB)

## Data Availability

Data available on request due to privacy/ethical restrictions.
