# Supplementary material for: Evaluation of oral health in 148 patients with systemic sclerosis—data from a prospective interdisciplinary monocentric cohort
Source: Rheumatol Int. 2024 Jun 14;44(8):1567–73. doi: 10.1007/s00296-024-05635-z (PMC11222211; doi:10.1007/s00296-024-05635-z)
Supplement: Supplementary file 1 — Supplementary file1 (DOCX 20 KB) [file 296_2024_5635_MOESM1_ESM.docx]

**Supplementary**

**Supplementary table 1** Characteristics of the patient cohort and results of the self-administered questionnaire (variables are highlighted in light grey). In case of missing values, percentage was calculated using the available number of patients.

| **Characteristic** | **all patients** | **female** | **male** |
| --- | --- | --- | --- |
| *Number of patients* | 148 | 111 | 37 |
| **Disease characteristics** |  |  |  |
| *Median disease duration in years (min-max)* | 6 (0-34) | 7 (0-34) | 4 (0-24) |
| *Patients with dcSSc, no. (%)* | 60 (41) | 47 (42) | 13 (35) |
| *SSc-associated autoantibody; no. (%)*  *Scl70*  *ACA*  *RNA Polymerase III*  *SSA / SSB* | 71 (48)  37 (25)  5 (3)  14 (9) | 21 (57)  35 (32)  3 (3)  12 (11) | 21 (56,8)  2 (5)  2 (5)  2 (5) |
| **Demographics** |  |  |  |
| *Median age in years (min-max)* | 53 (21-83) | 52 (22-80) | 54 (21-83) |
| *BMI* [kg/m²]; n (%)  <18.5 kg/m² (underweight)  18.5 – 25 kg/m² (normal)  25 – 30 kg/m² (overweight)  > 30 kg/m² (obese) | 10 (7)  90 (61)  36 (24)  12 (8) | 8 (7)  73 (66)  21 (19)  9 (8) | 2 (5)  17 (46)  15 (41)  3 (8) |
| *Years of formal education*  *<10 years*  *>10 years* | 65 (45)  80 (55) | 45 (42)  63 (58) | 20 (54)  17 (46) |
| *Smoking habits*; n (%)  *Current smoker*  *Never*  *Former smoker* | 13 (9)  89 (61)  44(30) | 8 (7)  73 (66)  29 (26) | 5 (14)  16 (44)  15 (42) |
| *Level of alcohol consumption*  *None*  *occasionally*  *2-3 times per week*  *1-2 times per day*  *>2 times per day* | 62 (43)  64 (44)  15 (10)  3 (2)  1 (1) | 48 (44)  47 (44)  10 (9)  2 (2)  1 (1) | 14 (38)  17 (46)  5 (14)  1 (3)  0 (0) |
| **Comorbidities; n (%) ^1^** |  |  |  |
| *Diabetes mellitus*  *Malignancy*  *Lactose intolerance*  *Dyslipidaemia*  *Heart failure* | 6  5  10  7  10 | 5  5  10  4  6 | 1  0  0  3  4 |
| **Oral hygiene** |  |  |  |
| *Frequency of dental visits*  *Never*  *2x per year*  *1x per year*  *In case of symptoms*  *Every two years* | 5 (3)  88 (60)  42 (28)  9 (6)  3 (2) | 2 (2)  63 (57)  36 (32)  6 (5)  3 (3) | 3 (8)  25 (68)  6 (16)  3 (8)  0 (0) |
| *Professional tooth cleaning*  *Never*  *1x per year*  *2x per year* | 43 (30)  44 (31)  54 (38) | 33 (31)  30 (29)  42 (40) | 10 (28)  14 (39)  12 (33) |
| *Toothbrushing frequency per day*  *Never*  *1x*  *2x*  *3x*  *>3x* | 2 (1)  17 (12)  109 (74)  16 (11)  2 (1) | 0 (0)  13 (12)  85 (77)  10 (9)  1 (1) | 2 (5)  4 (11)  24 (65)  6 (16)  1 (3) |
| *Usage of electronic toothbrush*  *Yes*  *No* | 84 (57)  61 (41) | 65 (59)  43 (39) | 19 (51)  18 (49) |
| *Usage of interdental care devices^2^*  *Never*  *Daily*  *4-6x per week*  *1-3x per week* | 51 (35)  53 (36)  8 (5)  22 (16) | 33 (30)  41 (37)  7 (6)  20 (18) | 18 (47)  12 (32)  1 (3)  2 (5) |
| *Usage of mouthwash*  *Never*  *Daily*  *4-6x per week*  *1-3x per week* | 95 (64)  28 (19)  3 (2)  18 (12) | 71 (64)  19 (17)  3 (3)  15 (14) | 24 (65)  9 (24)  0 (0)  3 (8) |
| *Problems opening the mouth*  *No*  *Yes, due to reduced mouth opening*  *Yes, due to the tearing of the corner of the mouth*  *Yes, due to both* | 67 (45)  63 (43)  2 (1)  14 (10) | 46 (41)  50 (45)  1 (1)  13 (12) | 21 (57)  13 (35)  1 (3)  1 (3) |
| *Difficulty holding toothbrush*  *No*  *Yes, due to reduced dexterity*  *Yes, due to pain*  *Yes, due to both* | 104 (70)  22 (15)  10 (7)  11 (7) | 80 (72)  12 (11)  9 (8)  9 (8) | 24 (65)  10 (27)  1 (3)  2 (5) |
| *Self-reported oral sicca symptoms; median (min-max)*  *on a visual analogue scale [0-10]* | 3.8 (0-10) | 5 (0-10) | 1.0 (0-8) |
| *Drinking while eating*  *Never*  *Rarely*  *Occasionally*  *Frequently*  *Regularly* | 56 (38)  36 (24)  32 (22)  10 (7)  9 (6) | 38 (34)  28 (25)  23 (21)  9 (8)  8 (7) | 18 (49)  8 (22)  9 (24)  1 (3)  1 (3) |
| *Difficulty swallowing food*  *Never*  *Rarely*  *Occasionally*  *Frequently*  *Regularly* | 45 (30,4)  41 (27,7)  38 (25,7)  12 (8,1)  10 (6,8) | 32 (29)  29 (26)  31 (28)  10 (9)  7 (6) | 13 (35,1)  12 (32,4)  7 (18,9)  2 (5,4)  3 (8,1) |
| **Oral health** |  |  |  |
| *Dental/mouth hygiene impairment; median (min-max)*  *on a visual analogue scale [0-10]* | 2 (0-10) | 2 (0-10) | 1 (0-9) |
| *Missing teeth*  *No own teeth*  *Some*  *No missing teeth* | 10 (7)  78 (53)  55 (37) | 6 (5)  60 (54)  41 (37) | 4 (11)  18 (49)  14 (38) |
| *Temporomandibular joint disorder*  *Yes*  *No* | 40 (27)  106 (72) | 35 (14)  72 (67) | 5 (14)  32 (87) |
| *Self-reported presence of mobile teeth*  *Yes*  *No* | 25 (17)  116 (78) | 18 (16)  88 (79) | 7 (19)  28 (76) |
| *Self-reported presence of gingival bleeding*  *No*  *Occasionally*  *Yes* | 81 (55)  53 (36)  12 (8) | 60 (54)  40 (36)  9 (8) | 21 (57)  13 (35)  3 (8) |

Abbreviations: SSc, systemic sclerosis; BMI, body mass index; dcSSc, diffuse cutaneous SSc; Scl70, anti-topoisomerase; ACA, anti-centromere; min, minimum; max, maximum. ^1^no reported percentage due to missing data in n=27 female and n=5 male patients. ^2^ data missing from n=10 female and n=4 male patients.
